# Application of an *in vitro*-amplification assay as a novel pre-screening test for compounds inhibiting the aggregation of prion protein scrapie

**DOI:** 10.1038/srep28711

**Published:** 2016-07-07

**Authors:** Matthias Schmitz, Maria Cramm, Franc Llorens, Niccolò Candelise, Dominik Müller-Cramm, Daniela Varges, Walter J. Schulz-Schaeffer, Saima Zafar, Inga Zerr

**Affiliations:** 1 Department of Neurology, University Medical Center Göttingen and German Center for Neurodegenerative Diseases (DZNE)-Göttingen campus, Göttingen, Germany; 2 Department of Neuropathology, Georg-August University, 37075 Göttingen, Germany

## Abstract

*In vitro* amplification assays, such as real-time quaking-induced conversion (RT-QuIC) are used to detect aggregation activity of misfolded prion protein (PrP) in brain, cerebrospinal fluid (CSF) and urine samples from patients with a prion disease. We believe that the method also has a much broader application spectrum. In the present study, we applied RT-QuIC as a pre-screening test for substances that potentially inhibit the aggregation process of the cellular PrP (PrP^C^) to proteinase (PK)-resistant PrP^res^. We chose doxycycline as the test substance as it has been tested successfully in animal models and proposed in clinical studies as a therapeutic for prion diseases. The RT-QuIC-reaction was seeded with brain tissue or CSF from sCJD patients and doxycycline was then added in different concentrations as well as at different time points. In both experiments, we observed a dose- and time-dependent inhibition of the RT-QuIC seeding response and a decrease of PK resistant PrP^res^ when doxycycline was added. In contrast, ampicillin or sucrose had no effect on the RT-QuIC seeding response. Our study is the first to apply RT-QuIC as a pre-screening assay for compounds inhibiting the PrP aggregation *in vitro* and confirms that doxycycline is an efficient inhibitor of the PrP aggregation process in RT-QuIC analysis.

Transmissible spongiform encephalopathies (TSE) are a group of neurodegenerative disorders, such as Creutzfeldt-Jakob disease (CJD), which are characterised by the conversion and accumulation of a disease-associated, proteinase K (PK)-resistant, misfolded isoform of the cellular prion protein (PrP^C^) called scrapie prion protein (PrP^Sc^). TSEs include spontaneously (sporadic), genetic and acquired forms of CJD. In humans, sporadic CJD (sCJD) is the most common form of prion disease, followed by genetic CJD (gCJD) and iatrogenic CJD (iCJD). TSEs are non-curable neurodegenerative disorders with an average survival time of 6 months after onset. According to the protein-only hypothesis^1^, PrP^Sc^ (also termed PrP^res^, PrP^CJD^) is the principal causative agent which is composed of an abnormally misfolded, multimeric, and PK-resistant form of the host’s PrP, which can induce, or seed, its own propagation by recruiting and converting the cellular and protease-sensitive prion protein, PrP^C^.

Various *in vitro* conversion assays, such as protein misfolding cyclic amplification (PMCA), or real-time quaking-induced conversion (RT-QuIC), use the high aggregation and seeding activity to amplify miniscule amounts of PrP^res^ to a detectable level.

The adaptation of *in vitro* amplification systems to detect misfolded PrP^res^ in human cerebrospinal fluid (CSF) as a pre-mortem test is an important innovation, because it permits the study of the aggregation processes of misfolded PrP^res^ *in vitro* for the first time^2^,^3^. RT-QuIC analysis uses recombinant prion protein (recPrP) as a substrate to amplify small amounts of a misfolded PrP^res^ seed in human CSF or brain tissue to a detectable level. Aggregated PrP^res^ can be monitored in real-time by fluorescence dye analysis using a fluorescent plate reader. The kinetic of the signal detection is used to evaluate the efficiency of the reaction.

Until now RT-QuIC has been applied in routine prion disease diagnosis and to study the seeding of different prion strains^4^,^5^,^6^,^7^,^8^. However, this methodology has a huge analytical potential beyond the already known applications. Since PrP^res^ is the main causative agent of prion diseases, most potential substances tested for therapy are targeted against PrP^res^, against the conversion process of PrP^C^ in PrP^res^ or against the PrP^res^ aggregation.

The tetracycline, doxycycline, has already been reported to bind to PrP and to decrease the amount of infectious PrP^res^ in animal models^9^,^10^,^11^. Doxycycline extended the survival time of scrapie-infected hamsters^10^; however, one clinical trial study failed, probably because treatment started at later stages^12^.

Until now there has been no treatment available for prion diseases, although a number of compounds have shown positive effects in animal models^9^,^10^,^11^,^12^. One of the reasons for delay in translational applications of various compounds in humans is that an easy and reproducible test system to investigate the effect of substances on the conversion and aggregation process of human PrP has been lacking.

In the present study, we applied the RT-QuIC assay to analyse the impact of doxycycline on the conversion and aggregation of PrP^res^ by adding doxycycline in different concentrations and at different times to the reaction mixture, which was seeded with brain tissue or CSF from CJD and control patients. We monitored thioflavin-T (Th-T) fluorescence and the kinetic curves obtained during aggregation of recPrP in the presence and absence of the drug. The fluorescence signals of the RT-QuIC reactions were characterised by their areas under the curve (AUC). Finally, we compared the amount of PK resistant PrP^res^ after RT-QuIC analysis and in brain tissue in dependence from doxycycline treatment.

## Materials and Methods

### Patients and trials

Patient CSF samples were collected through routine activities of the German National Prion Disease Surveillance Center, based at the University Medical Center Göttingen. Brain samples (frontal cortex) were obtained from ten sporadic CJD (sCJD) patients typed as codon 129 MM1 and VV2. PRNP mutation was excluded by sequencing of PRNP. CSF samples were taken from 19 definite sCJD patients, typed as codon 129 MM1 or VV2 and 12 control patients without prion disease. Patients used as controls exhibited either a clinically or pathologically definitive alternative diagnosis.

### Samples

Before use, brain and CSF samples were stored at −80 °C. Blood-contaminated CSF samples were excluded. The study conformed to the Code of Ethics of the World Medical Association, all study participants or their legal next of kin gave informed consent and the study was approved by the local ethics committee in Göttingen (No. 24/8/12). All samples were anonymised with regard to personal data. Brain samples, obtained from the Department of Neuropathology, University Medical Center, were homogenised (10% w/v) in a lysis buffer containing 1% SDS (Sigma), 100 mM Tris/HCl (Sigma-Aldrich), 100 mM sodium chloride (Roth) and 100 mM EDTA (Sigma-Aldrich) and then diluted to 10^−3^ in 1x PBS (Biochrom).

### RT-QuIC analysis

The RT-QuIC was performed as reported before^6^,^8^. 85 μL of reaction buffer (consisting of 5xPBS (pH 6.9), 170 mM sodium chloride (SAFC Biosciences), 1 mM EDTA (Sigma-Aldrich), 10 μM Th-T (Sigma-Aldrich), and 0.1 mg/mL recPrP^C^ (Thermo Fisher Scientific/Prionics) was seeded with 15 μL CSF or 15 μL of brain homogenate at a dilution of 10^−3^ to a final volume of 100 μL in a 96-well black optical bottom plate (Fisher-Scientific). Each patient sample (brain and CSF) was analysed in triplicate. Prepared plates were sealed with sealing tape (Nunc/Sigma-Aldrich) and incubated in a FLUO Star OPTIMA plate reader (BMG Labtech) at 42 °C for 80 hours with intermittent quaking cycles, consisting of one-minute double-orbital quaking at 600 rpm followed by a one-minute incubation break. Beta-sheet formation kinetic was determined by measuring the Th-T fluorescence signal (450 nm excitation and 480 nm emission) every 30 minutes in relative fluorescence units (rfu).

### Expression and purification of recombinant hamster-sheep PrP

For RT-QuIC, we used chimeric recPrP^C^, which consists of the Syrian hamster PrP (residues 23 to 137) followed by sheep PrP (residues 141 to 234 of the R_154_ Q_171_ polymorphic haplotype), as described before^8^,^13^. The recPrP was synthesised according to a previous reported method^2^.

### Substance treatment

To analyse the influence of doxycycline (Sigma-Aldrich), ampicillin (Fluka), sucrose (Sigma-Aldrich) and bovine serum albumine (BSA) (Sigma-Aldrich) on the RT-QuIC seeding response, we resolved all substances in RT-QuIC reaction buffer. To investigate the influence of the time point after a substance was added to the reaction on the RT-QuIC response, we added 5 μL of 10 mM doxycycline, ampicillin or sucrose to the reaction mix (final concentration of 0.5 mM). The treatment occurred either directly or after different time points (0 h, or at 24, 48 or 72 h) after the RT-QuIC has been started.

### Membrane adsorption assay (MAA)

Brain tissue was homogenized 1:10 in in PBS including 0.5% desoxycholate (Fluka). DNA was removed by DNAse I [5 μg enzyme/mg brain tissue (Applichem)] for 30 min at 37 °C. The membrane adsorbtion assay was performed as described previously^14^. In detail, brain homogenates were digested by proteinase K [50 μg/mL final concentration (Sigma-Aldrich) in PK-buffer buffer [TBS containing 0.1% Brij, (Sigma Aldrich)] at 37 °C for 35 min. A nitrocellulose membrane (pore size 0.45 μm, Bio-Rad) was soaked with 30 mL 1:10 in distilled water diluted Roti^®^-Block (Roth) for 10 min. The samples were sucked through the membrane in a Bio-Rad Microfiltration Apparatus (Bio-Dot SF) using a diaphragm pump (type MD1, Vacuubrand). Slots were rinsed with 200 μL 0.1% desoxycholate before and after the application of gradually diluted samples in volumes of 180–360 μL.

The membrane was decontaminated in 4 M guanidine thiocyanate (GdnSCN, Amresco, Solon, Ohio) for 30 min, which is also crucial for epitope retrieval. Blocking steps with 3% hydrogen peroxide (Applichem, Darmstadt, Germany) and casein 0.2% (I-Block, Applied Biosystems, Foster City, California) were performed before the primary antibody mAb 3F4 was applied at 1:3000 for 60 min at room temperature. Antigen-antibody reaction was visualized on x-ray films using an HRP-coupled goat anti-mouse antibody (1:1000, Dako, Carpintera, California) and a chemiluminescent substrate (Super Signal Femto West, Perbio, Erembodegem, Belgium).

### Proteinase K (PK) treatment

We followed a modified protocol published by *Orrú et al.*^15^. RT-QuIC products were digested with a mild PK concentration of 5 μg/mL for 60 min at 37 °C.

PK was inactivated by heating the samples at 65 °C for 30 Min.

### SDS-PAGE and Immunoblotting

Western blotting was performed according to a protocol as described before^16^.

We used the PrP specific antibody SAF32 (SPI-Bio, Montigny Le Bretonneux Paris, France) in a dilution 1:500. Briefly, after PK-treatment samples were separated by sodium dodecyl sulfate-polyacrylamide gel electrophoresis (SDS-PAGE) (15% w/v polyacrylamide) and transferred to polyvinylidene difluoride (PVDF) Hydrobond-P membranes (Amersham). Protein-labeled PVDF-membranes were probed with SAF32 overnight at 4 °C. Visualization of protein bands occurred by using an enhanced chemiluminescence (ECL) detection system solution using Chemicon system (Bio-Rad).

### Statistical analysis

AUCs were calculated using Excel 2010 and for convenience divided through 1000 and groups were compared in GraphPad Prism 6.05 (GraphPad Software), using *1-way ANOVA and Tukey’s post hoc test*. Graphically means and standard errors are shown for each group.

## Results

### Influence of doxycycline on the RT-QuIC seeding response seeded with brain tissue of sCJD patients

**Figure 1:**
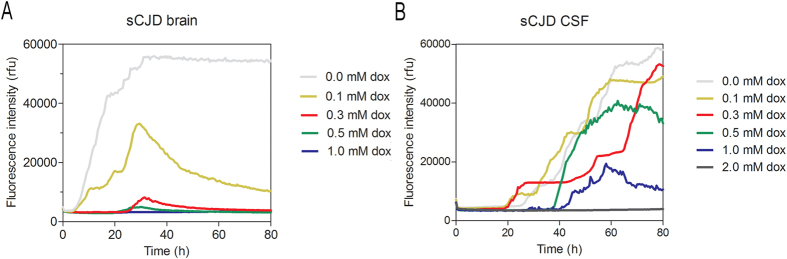
Impact of doxycycline on the RT-QuIC seeding response of sCJD brain and CSF samples. (**A**) RT-QuIC reactions, seeded with brain tissue (10% v/w) from sCJD (MM1) patients (n = 5) in a dilution of 10^−3^ and (**B**) CSF from sCJD (MM1) patients (n = 14), were performed with different concentrations of doxycycline. The mean seeding response time course curves are shown.

To investigate whether doxycycline may influence the RT-QuIC seeding response, we added doxycycline in different concentrations (0–1 mM) to RT-QuIC reactions seeded with brain tissue from sCJD (MM1) patients (10% v/w) in a dilution of 10^−3^ (Fig. 1A).

Fluorescence intensities were measured in rfu. RT-QuIC reactions without doxycycline were used as a reference (Fig. 1A). To exclude any PRNP codon 129 genotype or PrP^Sc^ type effects on the RT-QuIC seeding response^8^, our focus was on MM1 sCJD cases only. For a quantitative assessment, we defined the AUC as the seeding parameter of interest.

**Figure 2:**
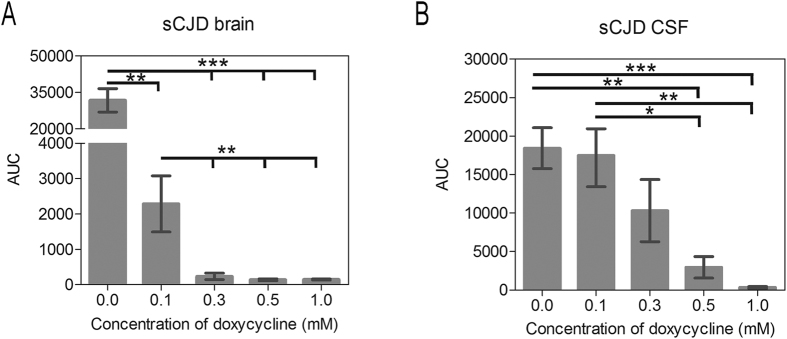
Impact of doxycycline on the AUC of RT-QuIC seeding response of sCJD brain and CSF samples. The AUC was defined as the parameter of interest to characterise the RT-QuIC seeding response (**A**) RT-QuIC reactions, seeded with brain tissue in a dilution of 10^−3^ from sCJD (MM1) patients (n = 5) were treated with different concentrations (0.0, 0.1, 0.3, 0.5 and 1.0 mM) of doxycycline. Doxycycline concentrations of 0.1 mM or higher resulted in an inhibition of the seeding of PrP. (**B**) The inhibition of the PrP conversion via doxycycline was also obtained in RT-QuIC reactions seeded with CSF from sCJD (MM1) patients (n = 14). Doxycycline concentrations of 0.5 mM or higher resulted in an inhibition of the seeding of PrP. Non-treated reactions were used as a reference (0.0). A p-value: <0.001 as extremely significant (***), <0.01 as very significant (**), <0.05 as significant (*) and ≥0.05 as not significant (ns).

We found that doxycycline inhibited the RT-QuIC seeding response in a dose-dependent manner. The seeding response was significantly decreased when doxycycline was applied in a concentration of 0.1 mM, and a complete inhibition of the seeding response was achieved after application of 1 mM of doxycycline (Figs 1A and 2A).

### Influence of doxycycline on the RT-QuIC seeding response seeded with CSF of sCJD patients

To determine whether doxycycline might also influence the PrP seeding response in CSF, we added doxycycline to RT-QuIC reactions seeded with CSF from sCJD (MM1) patients.

Doxycycline was added in different concentrations (0–2 mM) as well as at different time points (0, 24, 48 and 72 h) to the RT-QuIC reaction. For a quantitative comparison, we defined the AUC as the seeding parameter of interest.

We found that doxycycline inhibited the RT-QuIC seeding response in a dose-dependent manner. A decrease in the RT-QuIC seeding response was obtained when doxycycline was applied in a concentration of 0.5 mM and a complete inhibition of the seeding response was achieved after application of 2 mM of doxycycline (Figs 1B and 2B). Interestingly, the inhibitory effect of doxycycline was more efficient on brain seeded reactions than when using a CSF seed (Figs 1A,B and 2A,B).

In order to analyse the effect of doxycycline on the time course of the PrP seeding process, we added doxycycline at different time points (0, 24, 48 and 72 h) to the RT-QuIC reaction seeded with CSF from sCJD (MM1) patients. As a reference we used untreated reactions seeded with CSF from the same patients.

**Figure 3:**
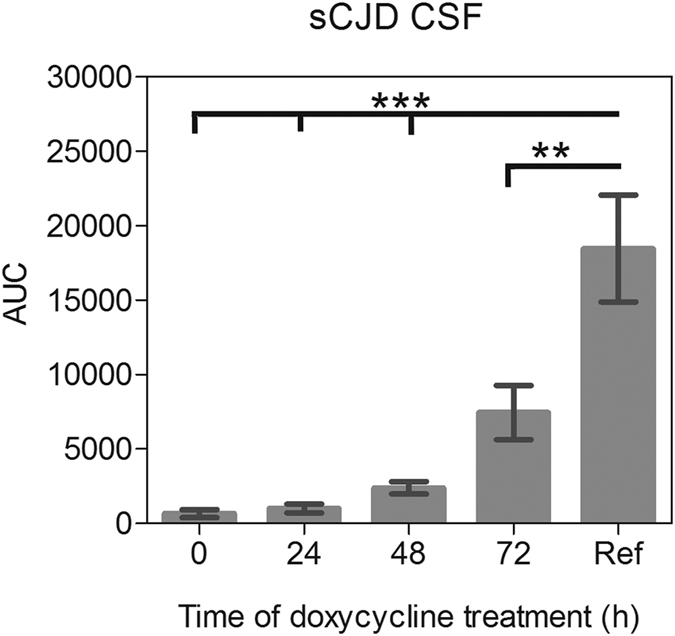
Time-dependent treatment of RT-QuIC reactions with doxycycline. PrP-seeding activity was measured in sCJD (MM1) CSF samples (n = 14), treated with doxycycline at different time points during the RT-QuIC analysis. 0.5 mM doxycycline was added either at the beginning of the run (t = 0) or at 24 (t = 24), 48 (t = 48) or 72 hours (t = 72) after the run had been started. Early addition of doxycycline resulted in a complete inhibition of PrP-seeding activity. Non-treated reactions were used as a reference (Ref). A p-value: <0.001 as extremely significant (***), <0.01 as very significant (**), <0.05 as significant (*) and ≥0.05 as not significant (ns).

When doxycycline (0.5 mM) was added within the first 24 h, we observed no seeding activity of PrP (signal on control level). Interestingly, when doxycycline was added at later times after reaction had started (after 48 or 72 h) the RT-QuIC seeding response was reduced compared to the untreated controls (Fig. 3).

Control experiments were performed by adding the antibiotic ampicillin and sucrose, in different concentrations (0–1 mM) and at different times (0–72 h), to the RT-QuIC reaction seeded with CSF from sCJD (MM1) patients.

Neither ampicillin nor sucrose affected the RT-QuIC response in sCJD patients (Supplement 1A–D). CSF from non-prion disease control-seeded reactions remained below 10,000 rfu (cut-off), regardless of the doxycycline, ampicillin or sucrose concentration (0–1 mM) and the time of treatment (0–72 h) (Supplement 2A–C and 3A–C). Moreover, we excluded a pH-dependent effect on the RT-QuIC seeding response, which decreased marginally from 7.1 to 7.0 when doxycycline, ampicillin or sucrose was added.

In another control experiment, we analysed the impact of bovine serum albumin (BSA), which is known to interact with Th-T^17^, on the RT-QuIC seeding response. As seed we used CSF from sCJD MM1 patients. Even though a BSA treatment revealed no significant influence on the AUC value of the RT-QuIC response (Supplement 4A), it causes a characteristic change of the kinetic of the RT-QuIC response when compared to sucrose or ampicillin. The unspecific binding of BSA to Th-T resulted in a spontaneous signal increase at time point 0 but it prevented a seeding reaction of PrP (Supplement 4B–D).

### Impact of doxycycline on other sCJD subtypes

Moreover, we were interested to know whether the doxycycline effect might be restricted to a certain PRNP codon 129 genotype or to a specific sCJD subtype. We analysed doxycycline treated RT-QuIC reactions, which were seeded with brain and CSF derived from sCJD VV2 patients. The obtained data indicated that the doxycycline effect is neither restricted to the codon 129 genotype nor to the prion type (Supplement 5A,B).

### Effect of shaking on the seeding activity after doxycycline treatment

**Figure 4:**
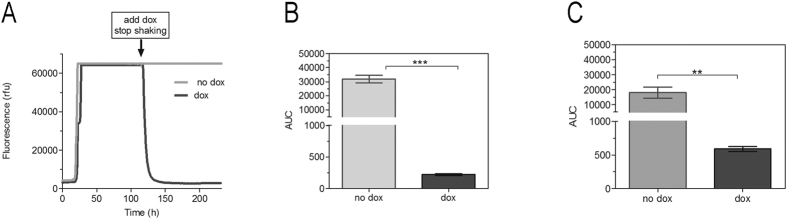
Impact of shaking on the efficiency of doxycycline treatment. RT-QuIC reactions were seeded with brain tissue or CSF from sCJD MM1 patients (n = 5 each). After 55% of the total experiment duration of 233 h, doxycycline was added directly into the RT-QuIC reaction (1 mM final concentration) and following the samples were incubated at 42 °C without shaking until the end or the run (**A**, schematic illustration). Quantification of the RT-QuIC signalling response after doxycycline treatment revealed a significant decrease in the AUC in brain (**B**) as well as in CSF-seeded reactions (**C**). Non-doxycycline-treated reactions were used as a reference (no dox). A p-value: <0.001 as extremely significant (***), <0.01 as very significant (**), <0.05 as significant (*) and ≥0.05 as not significant (ns).

To discover whether shaking would influence the anti-prion activity of doxycycline, we started an experiment with two phases. In the first phase, RT-QuIC reactions were seeded with brain tissue (10% w/v, 10^−3^ in PBS) or CSF from sCJD patients. After 55% of the total experiment duration of 233 hours, we obtained an increase in signal intensity of >10,000 rfu (typical for sCJD); in the second phase we added doxycycline (1 mM final concentration) directly into the RT-QuIC reaction. From the time of doxycycline treatment, we stopped the shaking process and incubated the samples at 42 °C until the end of the run as illustrated in Fig. 4A. Subsequently, we studied the effect of doxycycline on the PrP seeding in the RT-QuIC reactions by calculating the AUC. Interestingly, we observed a marked decrease in the RT-QuIC signal response after doxycycline treatment of sCJD brain and CSF seeded reactions, indicating that shaking is not required for an anti-prion effect of doxycycline on the RT-QuIC seeding response (Fig. 4B,C).

### Detection of PK-resistant PrP aggregates using a membrane adsorption assay

**Figure 5:**
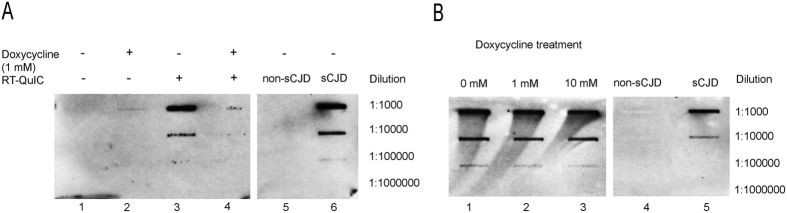
Detection of PK-resistant PrP via membrane adsorption assay. Samples were incubated with PK and subjected in different dilutions (1:1000 to 1:1000000) to a membrane adsorption assay. PK-resistant PrP^res^ was detected via 3F4 staining. (**A**) Lanes 1–4 show the filtation of RT-QuIC reaction mix seeded with brain homogenate from sCJD patients (MM1) (diluted 10^−3^) before and after the RT-QuIC run. Treatment with doxycycline (1 mM) reduced the amount of PK resistant PrP after RT-QuIC significantly (lane 4) compared to the untreated reaction (lane 3). (**B**) Brain homogenates from sCJD MM1 patients were incubated with different doxycycline concentrations (0–10 mM) and analysed via membrane absorption assay. Lanes 1–3 indicate that the amount of PK-resistant PrP remained unchanged after doxycycline treatment. No PK-resistant PrP was detected in the negative control consisting of brain homogenate (10%) from patients without prion disease (lane 4). Brain homogenates (10%) from sCJD MM1 patients revealed PK resistant PrP (lane 5). For confirmation we repeated the experiment three times with n = 3 patients.

To investigate the effect of doxycycline on the amount of PK-resistant PrP aggregates, we filtered PK resistant PrP aggregates of RT-QuIC reactions seeded with sCJD (MM1) brain homogenates. Interestingly, we observed that a doxycycline (1 mM) treatment reduced PK-resistant PrP in the RT-QuIC reaction compared to untreated reactions (Fig. 5A). This result underlines that the Th-T signal in the RT-QuIC correlates with the amount of aggregated and PK resistant PrP, which could be confirmed when we analysed the amount of PK resistant PrP after 24 h and 72 h after doxycycline treatment (Supplement 6A,B). However, when we treated brain homogenates from sCJD MM1 patients (without RT-QuIC) with doxycycline in different concentrations (0–10 mM), we observed no significant decrease in PK-resistant PrP^Sc^, indicating that doxycycline may not resolve existing PrP^Sc^ aggregates (Fig. 5B).

## Discussion

Over the last 30 years a number of potential prion disease treatments have been investigated in experimental prion disease models or in clinical trials^10^,^18^,^19^. Targets were substances that inhibit the conversion process, the aggregation and transmission process of PrP^Sc^. Unfortunately, most of the suggested compounds were not effective in treating the disease in humans. Drugs that have been tested often inhibited the propagation of PrP^Sc^ in cells or mice, but they were either not tested in humans because of their high toxicity or their failure to pass the blood brain barrier, or they showed a lack of a disease-relevant effect.

The translation of the experimental data to an application in humans has been hampered by the lack of suitable screening assays that mimic the disease relevant-conditions in humans. In our study, we used CSF and brain tissue from sCJD patients as seeds for the RT-QuIC reaction. Both seeds revealed an equal doxycycline effect, suggesting that less infectious sCJD-CSF is suitable for studying the potential effects of individual compounds on the RT-QuIC seeding response. The results of this study suggest that doxycycline binds to the seed particles in the RT-QuIC process and thus inhibits the transformation and the aggregation step.

Moreover, our findings propose the RT-QuIC assay as a pre-screening test for therapeutic substances targeting PrP^Sc^. Since not all substances inhibiting the RT-QuIC response are potential therapeutics of a prion disease, we suggest that the RT-QuIC will not replace animal experiments in therapeutic research but we consider this test as quite useful to exclude substances without any anti-prion effect.

### Doxycycline affects the seeding activity of PrP in sCJD patients

The RT-QuIC assay allows the *in vitro* amplification of PrP^res^ in human CSF and brain tissue. Until now this methodology has been applied for the purpose of diagnosing human prion diseases and studying prion type properties in seeding^4^,^5^,^6^,^7^,^8^. New data demonstrate that the assay is useful in studying the disease-specific biological characteristics of misfolded PrP^res^ directly using CSF samples from affected symptomatic patients^8^.

The huge potential of this methodology prompted us to test whether RT-QuIC might be suitable as a potential screening platform for substances that inhibit the conversion or aggregation process from PrP^C^ to PrP^res^. For proof-of-principle we used doxycycline as a compound, which has been shown to suppress the formation of infectious PrP^res^ and to prolong the survival time of infected hamsters^9^,^10^,^11^. We added doxycycline in different concentrations to the RT-QuIC reaction seeded with brain tissue or CSF from sCJD patients (MM1 and VV2). We observed a significant inhibition of the RT-QuIC seeding response, defined by the AUC, in doxycycline-treated reactions, suggesting that doxycycline inhibits the conversion process of PrP in the RT-QuIC reaction.

Furthermore, our data indicated that RT-QuIC reactions seeded with CSF are more resistant to the effects of doxycycline despite having a smaller AUC compared to brain seeded reactions. Possible explanations are (i) different amounts or (ii) different structures of PrP seeds in brain and CSF. In particular the structure of the seed may have an influence on the affinity to doxycycline which may result in different seeding capacity of both seeds.

The exact mechanism for how doxycycline exerts an anti-prion effect has not been studied in detail; however, some data are available. Doxycycline may directly bind to PrP and thus prevent the conversion process^10^,^20^. A suggested PrP binding site for tetracyclines is PrP amino acid 106–126^21^.

Control substances without any known anti-prion effect, such as ampicillin and sucrose, revealed no significant influence on the RT-QuIC signal response of samples seeded with CSF from sCJD patients.

Moreover, we tested BSA, which has no known anti-prion effect but it can interfere with Th-T^17^. BSA treatment showed no typical PrP-related inhibition of the RT-QuIC seeding reaction. The course of the kinetic curve started with an abnormal signal increase (>35000 rfu) at time point zero. During the RT-QuIC the signaling curve decreased, indicating an absence of a PrP seeding reaction. To interpret this observation, we can only speculate that the Th-T-BSA interaction hampered the detection PrP amyloids or that BSA additionally interfered with the PrP conversion and aggregation process in the RT-QuIC. To decipher the role of BSA, further experiments such as a testing of BSA in a PMCA assay or in a cell-based assay would be required.

To decrease the number of falsely classified anti-prion compounds, we suggest that substances with a known interference with Th-T or with other chemicals from the reaction buffer, which change the reaction conditions (such as a change of the pH or digest the rec PrP substrate), and which show abnormal RT-QuIC seeding kinetics should be excluded from analysis.

### Doxycycline reduces the RT-QuIC signal response without shaking

Studies in animal models revealed that the duration of doxycycline treatment had an impact on the survival time of infected animals^10^.

This prompted us to add doxycycline at different times to the RT-QuIC reaction and to study its influence on the RT-QuIC seeding response in connection with shaking. The idea was to measure (i) whether doxycycline only prevented the start of the PrP seeding, (ii) if it reduced the RT-QuIC seeding response signal and (iii) if shaking is required, (as this disaggregates PrP^res^ aggregates into smaller seeds).

Intriguingly, we observed a marked decrease in the brain- and CSF-seeded RT-QuIC signal response after doxycycline had been added, which indicates that doxycycline inhibits the conversion process of PrP.

When we studied the effect of shaking on the PrP aggregation process in doxycycline-treated reactions, we found the RT-QuIC seeding response reduced in both the shaken and non-shaken reactions. This indicates that doxycycline may not only bind to PrP, which originates from the disruption of oligomeric seed after shaking but also may directly influence the amount of PrP aggregates. To elucidate whether the Th-T-stained aggregated material is PK-resistant PrP^res^, we analysed the RT-QuIC product in a membrane adsorption assay which is more sensitive than a regular western blot.

The obtained data revealed a significantly reduced amount of PK-resistant PrP in doxycycline treated RT-QuIC reactions but not in brain tissue derived from sCJD patients.

Several explanations are possible. For example doxycycline may not only affect the conversion of PrP, it may also destabilize the structure of existing PrP aggregates which are different in the RT-QuIC reaction compared to brain tissue.

**Figure 6:**
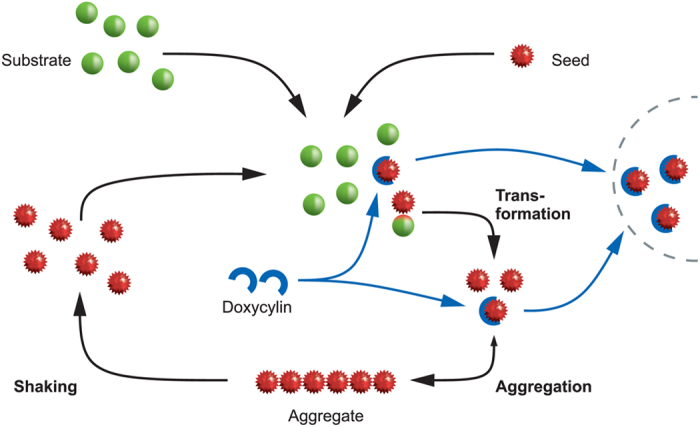
Schematic presentation of the RT-QuIC process with the potential points of action of doxycycline. The black arrows mark the steps of RT-QuIC. Misfolded PrP^res^ (seed) comes in contact with cellular prion protein (substrate), followed by a conformational change of the substrate to a more seed-like conformation (transformation). Newly generated seed particles aggregate and the aggregates are detected by a fluorescent dye (detection not shown). Then the aggregates are shaken to break them into seed particles again (shaking). The amount of seed particles and thus the fluorescent intensity increases by repeating this cycle frequently. The blue arrows mark the potential interference of the PrP conversion process via doxycycline. The results of this study suggest that doxycycline may destabilise aggregated and PK-resistant PrP.

We summarized the potential effect of doxycycline on the PrP conversion process in Fig. 6. A previous study has already demonstrated that doxycycline may revert infectious PK-resistant PrP^9^, which is in line with our observations.

### Considerations for translation into clinical application

Our observation that the effect of doxycycline inhibiting the PrP conversion depended on a concentration higher than 0.5 mM (222 μg/mL) gave rise to speculation on reasons why this compound has not been effective in clinical trials so far. First, the disease stage is extremely important − the administration at early or pre-symptomatic stages is likely to be most effective because of limited brain damage and a lower amount of infectious PrP^res^ in the brain. Secondly, the doxycycline concentration that reaches the brain is important. Even though it could be proven that doxycycline crosses the blood brain barrier, the median concentration of doxycycline decreases in CSF within 4 h to a concentration of 0.6 μg/mL (range 0.4 and 2.5 μg/mL) when 200 mg doxycycline were administered orally^22^. In most clinical trials, lower doses of doxycycline were used (100 mg daily). Also, doxycycline was administered with no regard to the gender or weight of the patients, which may explain the high range of doxycycline concentration and the efficiency. We propose that calculations for drug delivery should take the gender and weight of patients into account.

In conclusion, our study demonstrates that RT-QuIC is suitable as a testing platform to evaluate the potential efficiency of compounds to inhibit the PrP conversion process. In contrast to previous methods, this testing platform is suitable for use in biological fluids from symptomatic patients. This has a huge translational potential for drug screening for potential efficacy in treating these devastating prion diseases in humans.

## Additional Information

**How to cite this article**: Schmitz, M. *et al.* Application of an *in vitro*-amplification assay as a novel pre-screening test for compounds inhibiting the aggregation of prion protein scrapie. *Sci. Rep.* **6**, 28711; doi: 10.1038/srep28711 (2016).

## Supplementary Material

Supplementary Information
