# Supplementary material for: Application of an in vitro-amplification assay as a novel pre-screening test for compounds inhibiting the aggregation of prion protein scrapie
Source: Sci Rep. 2016 Jul 7;6:28711. doi: 10.1038/srep28711 (PMC4935936; doi:10.1038/srep28711)
Supplement: Supplementary Information [file srep28711-s1.pdf]

Application of an *in vitro*-amplification assay as a novel pre-screening test for compounds inhibiting the aggregation of prion protein scrapie

Matthias Schmitz[1#], Maria Cramm[1#], Franc Llorens[1], Niccolò Candelise[1], Dominik Müller-Cramm[1], Daniela Varges[1], Walter J. Schulz-Schaeffer[2], Saima Zafar[1] and Inga Zerr[1]

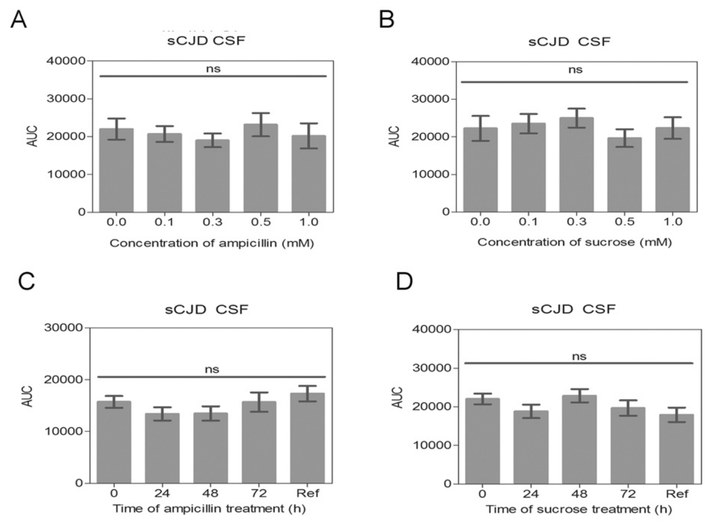

Supplement: 1

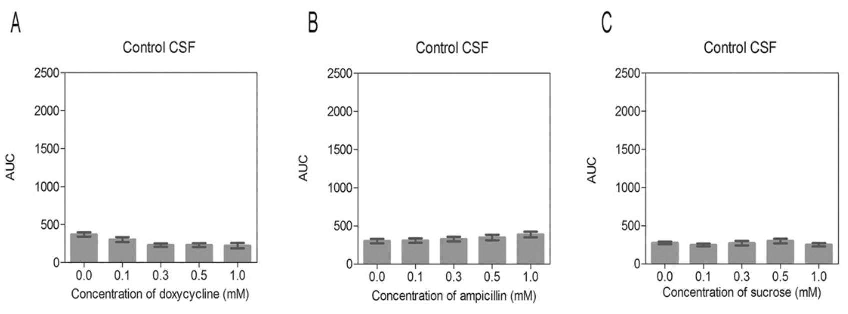

Supplement: 2

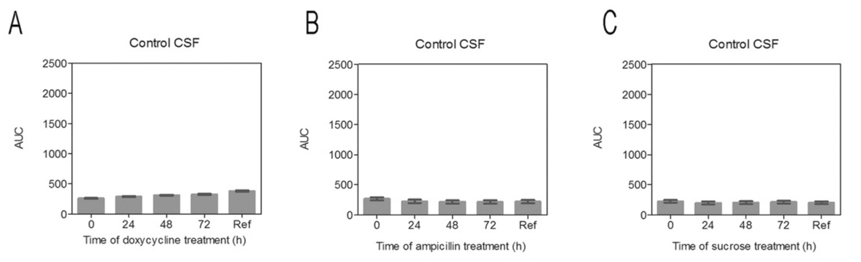

Supplement: 3

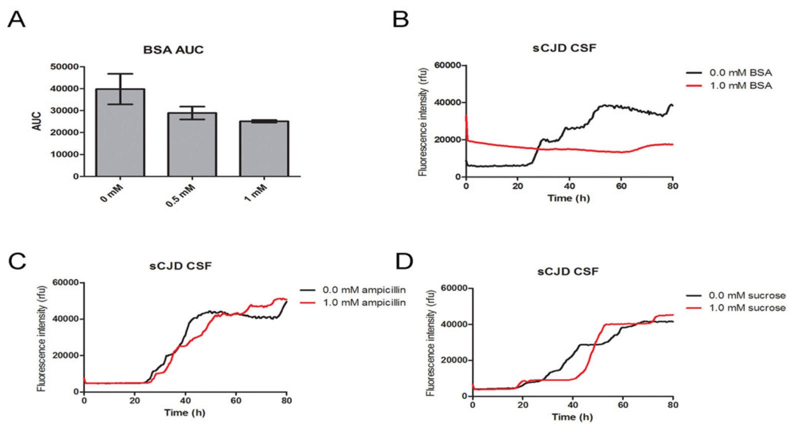

Supplement: 4

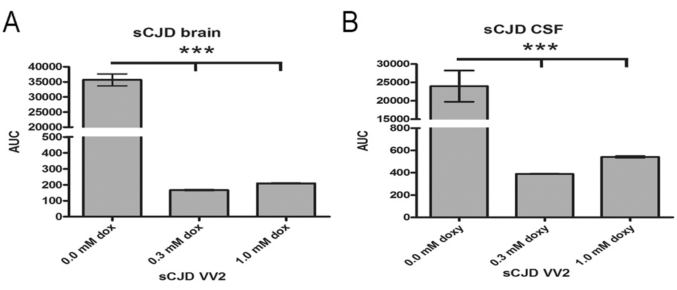

Supplement 5

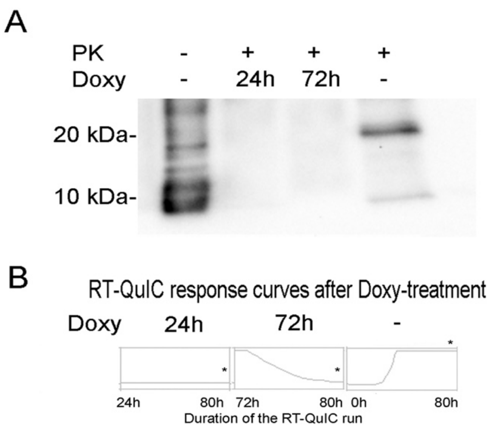

Supplement 6

Supplement 1: Impact of the duration and the concentration of ampicillin and sucrose treatment on the RT-QuIC response of sCJD CSF samples. (A) RT-QuIC reaction, seeded with CSF from sCJD (MM1) patients (n = 12) were treated with different concentrations of ampicillin (A) and sucrose (B) (0.1, 0.3, 0.5 and 1.0 mM). Non-treated reactions were used as reference (0.0). Neither ampicillin nor sucrose affected PrP-seeding activity as defined by AUC. PrP-seeding activity was measured in sCJD (MM1) CSF samples (n = 12) treated with ampicillin (C) and sucrose (D) at different times during the RT-QuIC analysis. 0.5 mM ampicillin and sucrose were added at the beginning of the run (t = 0) and 24 (t = 24), 48 (t = 48) as well as 72 hours (t = 72) after the run had been started showing no effect on the seeding of PrP. Non-treated reactions were used as reference (Ref). A p-value: < 0.001 as extremely significant (***), < 0.01 as very significant (**), < 0.05 as significant (*) and = 0.05 as not significant (ns).

Supplement 2: Impact of doxycycline-, ampicillin- and sucrose concentration on the RT-QuIC response of non-prion CSF samples. RT-QuIC reactions seeded with the CSF of control samples (n = 12) were treated with different concentrations of doxycycline (A), ampicillin (B) and sucrose (C) and analysed by RT-QuIC. Non-treated reactions were used as reference (0.0). All controls remained negative, regardless of the substance and its concentration.

Supplement 3: Impact of the time point of doxycycline, ampicillin and sucrose treatment on the RT-QuIC response of non-prion CSF samples. RT-QuIC reactions seeded with CSF of control samples (n = 12) were treated at different times with 0.5 mM doxycycline (A), ampicillin (B) and sucrose (C) and analysed by RT-QuIC. Non-treated reactions were used as reference (Ref). All controls remained negative, regardless of the substance and the time of administration.

Supplement 4: Impact of BSA treatment on the RT-QuIC signaling response of sCJD CSF samples. RT-QuIC reactions seeded with CSF of sCJDMM1 patients (n = 5) were treated with different concentrations of BSA. (A) Quantification of the RT-QuIC seeding response by calculating the area under the curve (AUC) values indicated no inhibitory effect of BSA (0.5 and 1 mM). (B-D) A comparison of the RT-QuIC signal kinetics of BSA, ampicillin and sucrose showed a spontaneous signal increase at time point zero in BSA (1 mM) treated reactions without a PrP seeding response.

Supplement 5: Influence of doxycycline on other sCJD subtypes
(A, B) RT-QuIC reactions, seeded either with brain tissue (A) (n=5) in a dilution of 10^{-3} or with CSF from sCJD (VV2) patients (n = 5) (B) were treated with different concentrations (0.0, 0.3 and 1.0 mM) of doxycycline. Calculation of the area under the curve (AUC) indicated a decrease of the RT-QuIC seeding after treatment with doxycycline.

Supplement 6: PK resistance of RT-QuIC products after treatment with doxycycline
(A) Western blot detection of PK resistant PrP derived from RT-QuIC reactions treated with doxycycline. (B) RT-QuIC curves indicated the PrP seeding reaction after doxycycline treatment. The time point when samples were treated with PK and analysed by Western blot was marked by an asterisk (*). The data indicated a correlation between the amount of PK resistant PrP and the Th-T signal in the RT-QuIC.
